# 

**Published:** 1895-06

**Authors:** 


*The following donations have been received since the publication of the list in March:*

*May 31st*, 1895.

| | |
|---|---|
| E. F. H. Burroughs (1) ... ... ... ... ... ... ... | 1 volume. |
| J. Michell Clarke, M.D. (2) ... ... ... ... ... ... | 7 volumes. |
| H. D. Rolleston, M.D. (3) ... ... ... ... ... ... | 1 volume. |
| Royal Academy of Medicine in Ireland (4) ... | 1 ,, |

### SIXTEENTH LIST OF BOOKS.

The titles of books mentioned in previous lists are not repeated.

The figures in brackets refer to the figures after the names of the donors, and show by whom the volumes were presented. The books to which no such figures are attached have either been bought from the Library Fund or received through the *Journal.*

---

**Abercrombie, J.**... *Diseases of the Brain and the Spinal Cord.* 2nd. Ed. 1829

**Addison, R. Bright and T.** *Elements of the Practice of Medicine.* Vol. I. 1839

**Andronesco, G. I.** *Considérations sur les Ruptures de l'Utérus et du Vagin pendant l'Accouchement* ... ... ... ... ... ... ... 1895

*Arctic Expeditions in search of Sir J. Franklin and the crews of H.M.S. "Erebus" and "Terror," Further papers relative to the recent* ... ... ... 1855

**Attfield, J.**... ... *The Laws of Nature in relation to Health* ... ... ... 1887

| | | | |
|---|---|---|---|
| **Baildon, S.** | *Tea in Assam* | | 1877 |
| **Ballantyne, J. W.** | *The Diseases of the Fœtus.* Vol. II. | | 1895 |
| **Banting, W.** | *Letter on Corpulence* | 4th Ed. | 1875 |
| **Barry, A.** | *The Preservation of Body and Soul and Spirit* | | 1881 |
| **Beach, F.** | *Mentally Feeble Children* | | 1895 |
| **Beck, C.** | *Manual of Surgical Asepsis* | | 1895 |
| **Belville, J. H.** | *Manual of the Barometer* | 3rd Ed. | 1858 |
| *Bible, La Sainte* | | | [N.D.] |
| **Bigg, R. H.** | *Orthopragms of the Spine* | | 1880 |
| **Bond, H. J. H.** | *Analysis of an Elementary Course of Lectures on Pathology* | | 1866 |
| **Bradley, T.** | *A New Medical Dictionary* | | 1803 |
| **Brementhal, D.** | *De Obliquitate Uteri quo ad Formam et Situm* | | 1843 |
| **Bright and T. Addison, R.** | *Elements of the Practice of Medicine.* Vol. I. | | 1839 |
| **Brodie, C. G.** | *Dissections Illustrated.* Part IV. | | 1895 |
| *Catalogue of the Library of the Royal Medical and Chirurgical Society of London* | | | 1856 |
| **Chisholm, J. E. Pollock and J.** | *Medical Handbook of Life Assurance.* | 4th Ed. | 1895 |
| *Cholera and its Prevention, The* | | | 1883 |
| *Church Lads' Brigade Medical Staff Corps, Manual for the* | | | 1895 |
| **Clanny, W. R.** | *Hyperanthraxis; or the Cholera of Sunderland* | | 1832 |
| **Clark, J.** | *On the Diseases which prevail in Long Voyages to Hot Countries* | 2nd Ed. | 1792 |
| **Clark, S.** | *The Hygiene of the Army in India* | | 1864 |
| *Cost's System of Calisthenic Exercises* | | 3rd Ed. | 1859 |
| **Cumming, J.** | *A Syllabus of a Course of Chemical Lectures* | | 1834 |
| **Dana, J. D.** | *A System of Mineralogy* | 3rd Ed. | 1850 |
| **Daniell, J. F.** | *An Introduction to the Study of Chemical Philosophy.* | 2nd Ed. | 1843 |
| **Davies, A. M.** | *A Handbook of Hygiene* | | 1895 |
| **Davis, D. D.** | *Elements of Operative Midwifery* | | 1825 |
| **Day, G. E.** | *Chemistry in its Relations to Physiology and Medicine* | | 1860 |
| **Drejer, P.** | *Om Tvillinger* | | 1895 |
| **Dührssen, A.** | *A Manual of Gynæcological Practice.* (Tr. and Ed. by J. W. Taylor and F. Edge) | | 1895 |
| **Dernen, P. J.** | *De optima Methodo Versionis Foetus instituendae* | | 1831 |
| **Fitzroy, Rear-Admiral.** | *Barometer Manual* | | 1861 |
| **Floerken, A.** | *De Superfoetatione* | | 1830 |
| **Forstheim, A.** | *De Pertussi* | | 1846 |
| **Fox, T.** | *Skin Diseases of Parasitic Origin* | | 1866 |
| ,, | *Prurigo and Pediculosis or Phtheiriasis* | | 1870 |
| **Foxwell, A.** | *Essays in Heart and Lung Disease* | | 1895 |
| **Fürbringer, P.** | *Diseases of the Kidneys and Urinary Organs.* Vol. I. (Tr. by W. H. Gilbert) | | 1895 |
| **Gottschalk, J.** | *Observationes nonnullae obstetrico-statisticae* | | 1836 |
| **Graham and H. Watts, T.** | *Elements of Chemistry.* 2 vols. | 2nd Ed. | 1850-58 |

**Grandin and G. W. Jarman, E. H.** *Obstetric Surgery* ... ... ... ... 1895

**Hammick, J. T....** *The Acts relating to the Registration of Births, Deaths, and Marriages, &c.* ... ... ... ... ... ... ... 1875

**Hart, E.** [Ed.] ... *A Manual of Public Health* ... ... ... ... ... ... ... 1874

**Harvey, W.** ... *The Circulation of the Blood* ... ... ... ... ... ... ... 1894

**Hellier, J. B.** ... *Infancy and Infant-Rearing* ... ... ... ... ... ... 1895

**Henslow, J. S.** ... *Syllabus of Lectures on Botany* ... ... ... ... ... 1853

**Herschell, G.** ... *Indigestion....* ... ... ... ... ... ... ... ... ... 2nd Ed. 1895

**Hill, R. G.** ... ... *Lunacy: its past and its present* ... ... ... ... ... 1870

**Hofmann, A. W.** *Contributions to the History of the Phosphorus-Bases* ... 1861

**Hughes, W. J. Walsham and W. K.** *The Deformities of the Human Foot* 1895

*Hunterian Relics exhibited at the Royal College of Surgeons of England, Catalogue of the collection of* ... ... ... ... ... ... ... ... ... ... ... 1893

*Hutton Collection of Fossil Plants, Catalogue of the*... ... ... ... ... ... 1878

**Jameson, R.** ... *Manual of Mineralogy* ... ... ... ... ... ... ... ... 1821
**Jarman, E. H. Grandin and G. W.** *Obstetric Surgery* ... ... ... ... 1895
**Johnston, J. F. W.** *Catechism of Agricultural Chemistry and Geology.* 27th Ed. 1851
**Jussieu, A. De** ... *The Elements of Botany* (Tr. by J. H. Wilson) ... 1849

**Kennedy, A. B. W.** *Kinematic Models* ... ... ... ... ... ... ... ... ... 1876
**Kirchgaesser, F. C.** *Blennorrhoeae Renum et Vesicae urinariae Adumbratio pathologica et therapeutica* ... ... ... ... ... ... 1825
**Klein, H.** ... ... *Partus memorabiles in Clinico obstetricio Bonnensi nuper observati et Annotationibus epicriticis illustrati* 1842

**Liddon, H. P.** ... *Teaching and Healing* ... ... ... ... ... ... ... ... 1881
**Lindley, [J.]** ... *Descriptive Botany* ... ... ... ... ... ... ... ... ... 1858
**Loehr, J. J.** ... *De Partium Corporis humani Situ abnormi cum Animi Alienatione* ... ... ... ... ... ... ... ... ... ... 1828
*Logarithms, Tables of* ... ... ... ... ... ... ... ... ... ... ... ... ... ... 1860
**Lucas, J.** ... ... *The Chalk Water System.* (Ed. by J. Forrest)... ... 1877
**Lyons, R. D.** ... *A Treatise on Fever* ... ... ... ... ... ... ... ... ... 1861

**MacCormac, Sir W.** *Surgical Operations.* Part I. ... ... ... ... (2) 1885
**Marie, P.** ... ... *Lectures on Diseases of the Spinal Cord.* (Tr. by M. Lubbock) ... ... ... ... ... ... ... ... ... ... 1895
**Martindale and W. W. Westcott, W.** *The Extra Pharmacopœia.* 8th Ed. 1895
**Mead, R.** ... ... *A Discourse on the Plague.* ... ... ... ... 9th Ed. 1744
**Milton, J. L.** ... *The Hygiene of the Skin.* ... ... ... ... 3rd Ed. 1886
*Mineralogical Species, A Table of* ... ... ... ... ... ... ... ... ... ... 1833
**Molesworth, W. N.** *Plain Lectures on Astronomy.* ... ... ... 2nd Ed. 1864
**Moure, E. J.** ... *Du Catarrhe Naso-Pharyngien* ... ... ... ... ... 1895
**Murphy, S. F.** [Ed.] *Our Homes and how to make them Healthy* ... ... 1885
**Murrell, W.** ... *Clinical Lectures on the Prevention of Consumption* ... 1895

**Nightingale, Florence.** *Notes on Hospitals* ... ... ... ... ... ... ... ... 1859

**Nysten, P. H.** ... *Manuel Médical.* ... ... ... ... ... ... ... 2nd Ed. 1816

**Parker, L.** ... ... *On Syphilitic Diseases.* ... ... ... ... ... ... 2nd Ed. 1845
**Parkes, E. A.** ... *A Manual of Practical Hygiene.* ... ... ... 4th Ed. 187

**Parnajon, F. De** *Exercices sur le Cours complet de Grammaire Grecque* 1864

**Peters, E.** ... ... *De legitimo pariendi Tempore* ... ... ... ... ... ... ... 1850

*Pharmacopœia Collegii Regalis Medicorum Londinensis* ... ... ... ... [1788]

*Pharmacopœia in usum Nosocomii Beatæ Virginis Mariæ* ... ... ... ... ... 1858

*Pharmacopœia Nosocomii Middlesexensis* ... ... ... ... ... ... ... ... ... ... 1865

*Pharmacopœia Nosocomii Regalis Sancti Bartholomæi* ... ... ... ... ... ... 1861

*Pharmacopœia Nosocomii Regalis Sancti Thomæ* ... ... ... ... ... ... ... 1867

*Pharmacopœia in usum Nosocomii Westmonasteriensis* ... ... ... ... ... ... 1840

**Pollock and J. Chisholm, J. E.** *Medical Handbook of Life Assurance.* 4th Ed. 1895

**Power, D'A.** ... *The Surgical Diseases of Children* ... ... ... ... ... ... 1895

**Ramón y Cajal, S.** *Nuevo Concepto de la Histología de los Centros Nerviosos* 1893
**Richards, T.** [Ed.] *New South Wales in 1881* ... ... ... ... ... ... ... 1882
**Rolleston, H. D.** *The Goulstonian Lectures on the Suprarenal Bodies* (3) 1895
**Russell, P.** ... ... *A Treatise on the Plague.* 2 vols. ... ... ... ... 1791

**Sankey, W. H. O.** *Lectures on Mental Diseases* ... ... ... ... ... ... ... 1866

**Savage, T.** ... ... *Oöphorectomy.* No. II. ... ... ... ... ... ... ... ... 1881

**Scoffern, J.** ... *Outlines of Botany* ... ... ... ... ... ... ... ... ... [N.D.]

**Sedgwick, A.** ... *A Syllabus of a Course of Lectures on Geology.* 3rd Ed. 1837

**Shapter, T.** ... ... *The History of the Cholera in Exeter in 1832* ... ... 1849

**Sinnett, F.** ... ... *An Account of the Colony of South Australia* ... .. 1862

**Smith, E.** ... ... *Manual for Medical Officers of Health* ... ... 2nd Ed. 1874

**Spencer, W. H.** ... *Inhalation* ... ... ... ... ... ... ... ... ... ... ... ... 1895

**Squire, B.** ... ... *On Lupus Erythematosus* ... ... ... ... ... ... ... ... 1887

,, ... ... *On Lupus Vulgaris* ... ... ... ... ... ... ... ... ... ... ... 1888

**Squire, P.** ... ... *The Pharmacopœias of seventeen of the London Hospitals.* 2nd Ed. 1869

**Stevens, J.** ... ... *An Essay on treating Cholera in its second, or Asiatic, Stage* ... ... ... ... ... ... ... ... ... ... 3rd Ed. 1849

**Stone, W. H.** ... *Sound and Music* ... ... ... ... ... ... ... ... ... ... 1876

**Thomson, R. D.** ... *Dictionary of Chemistry* ... ... ... ... ... ... ... ... [N.D.]

**Thorne, W. B.** ... *Chronic Diseases of the Heart* ... ... ... ... ... ... ... 1895

**Thue, K.** ... ... *Bidrag til Plevritens Ætiologi* ... ... ... ... ... ... 1895

**Tyrrell, F.** ... ... *Diseases of the Eye.* 2 vols. ... ... ... ... ... (2) 1840

**Unger, C.** ... ... *Perforatio et Cephalotripsia collatae* ... ... ... ... 1840

**Vintras, A.** ... .. *Diabetes* ... ... ... ... ... ... ... .. ... ... ... 1895

*Vocabulary of Diseases, A.* (English-Chinese) ... ... ... ... ... ... 1894

**Walsham and W. K. Hughes, W. J.** *The Deformities of the Human Foot* 1895
**Watts, T. Graham and H.** *Elements of Chemistry.* 2nd Ed. 2 vols. 1850-58
**Webb, A.** ... ... *Pathologia Indica.* ... ... ... ... ... ... ... 2nd Ed. 1848
**Westcott, W. Martindale and W. W.** *The Extra Pharmacopœia.* 8th Ed. 1895
**Williamson, R. T.** *On the Relation of Diseases of the Spinal Cord to the Distribution and Lesions of the Spinal Blood Vessels* 1895
**Willis, R.** ... ... *A Syllabus of a Course of Experimental Lectures on the Principles of Mechanism* ... ... ... ... ... ... ... 1841
**Wright, J.** ... ... *Mushrooms for the Million.* ... ... ... ... 5th Ed. 1887

**Zurmeyer, E.** ... *De Morbis Fetus* ... ... ... ... ... ... ... ... ... 1832

### TRANSACTIONS, REPORTS, JOURNALS, &c.

Alienist and Neurologist, The... ... ... ... ... ... ... ... ...Vol. XV. 1894
American Practitioner and News, The... ... ... Vols. XVII., XVIII. 1894
Annali di Ostetricia e Ginecologia ... ... ... ... ... ... ... ... ... ... 1894
Archives Cliniques de Bordeaux ... ... ... ... ... ... ... Tome III. 1894
Archives of Pediatrics ... ... ... ... ... ... ... ... ... ... ... Vol. XI. 1894
Australasian Medical Gazette, The ... ... ... ... ... ... Vol. XIII. 1894
Bristol Medico-Chirurgical Journal, The ... ... ... ... Vol. XII. 1894
Bristol Port Sanitary District—Annual Report of the Medical Officers of Health for 1894 ... ... ... ... ... ... ... ... ... ... ... ... 1895
Bulletin de l'Académie de Médecine ... ... Tomes XXXI., XXXII. 1894
Bulletin de l'Académie royale de Médecine de Belgique Tome VIII. 1894
Clinical Journal, The ... ... ... ... ... ... ... ... ... ... Vol. V. 1895
Edinburgh Medical Journal ... ... ... ... (2) Vol. XXXIII, Part 2 1888
Epidemiological Society of London, Transactions of the. N.S. Vol. XIII. 1894
Hospital, The ... ... ... ... ... ... ... ... ... ... ... Vol. XVII. 1895
Hospitals—
King's College Hospital Reports ... ... ... ... ... Vol. I. 1895
Saint Bartholomew's Hospital Reports ... ... ... Vol. XXX. 1894
Index Medicus ... ... ... ... ... ... ... ... ... ... ... ... Vol. XVI. 1894
International Medical Magazine ... ... ... ... ... ... ... ... Vol. III. 1895
Johns Hopkins Hospital Bulletin, The... ... ... ... ... ... ... Vol. V. 1894
Library, The ... ... ... ... ... ... ... ... ... ... ... ... ... Vol. VI. 1894
Liverpool Medico-Chirurgical Journal, The ... ... ... Vol. XIV. 1894
Local Government Directory, Almanac and Guide, The ... ... ... ... 1886
Medical Annual, The ... ... ... ... ... ... ... ... ... ... ... ... ... 1895
Medical Bulletin, The ... ... ... ... ... ... ... ... ... Vol. XV. 1893
Medical Chronicle, The ... ... ... ... ... ... ... ... N.S. Vol. II. 1895
Medical Magazine, The ... ... ... ... .. ... ... ... ... ...Vol. III. 1895
National Association for the Promotion of Social Science, Transactions of the, 1884 ... ... ... ... ... ... ... ... ... ... ... ... ... ... 1885
New Zealand Medical Journal, The ... ... ... ... ... ... Vol. VII. 1894
Nouvelle Iconographie de la Salpêtrière ... ... ... ... Tome VII. 1894
Obstetrical Society of London, Transactions of the... ...Vol. XXXVI. 1895
Pathological Society of London, Transactions of the...(2) Vols. XLIII.—XLV. 1892-94
Revue Internationale de Médecine et de Chirurgie pratiques ... ... ... 1894
Rochdale, Borough of—Street and Building Regulations ... ... ... 1873
Royal Academy of Medicine in Ireland, Transactions of the. (4) Vol. XII. 1894
Royal Humane Society, Annual Report of the ... ... ... ... ... ... 1814
Royal Institution of Great Britain—List of Members, Officers, &c., with Report of the Visitors for 1861; 1865; 1866; 1868—72; 1874.
St. Louis Medical and Surgical Journal, The Vols. LXV.—LXVII. 1893-94
St. Petersburger Medicinische Wochenschrift ... ... ... ... ... ... 1894
Semana Médica, La ... ... ... ... ... ... ... ... ... ... ... ... ... (1) 1894
Universal Medical Journal, The ... ... ... ... ... N.S. Vol. II. 1894
Year-Book of the Scientific and Learned Societies ... ... ... ... ... 1895

### Local Medical Notes.

UNIVERSITY COLLEGE, BRISTOL.

On Feb. 20th the Council of University College, Bristol, conferred the title of Emeritus Professor of Midwifery on J. G. Swayne, M.D., on his resignation of the post of Lecturer on Midwifery in the Bristol Medical School, which he had held for fifty years. On the same day the Council conferred the title of Emeritus Professor of Surgery on Nelson C. Dobson, F.R.C.S., on his retirement from the post of Lecturer on Surgery.

J. E. Shaw, M.B. Edin., Physician to the Bristol Royal Infirmary, has been appointed Professor of Medicine, in place of R. Shingleton Smith, M.D. Lond., B.Sc., F.R.C.P., resigned.

The following students have passed the undermentioned Examinations:—

**University of London.**—Intermediate Examination for the Degree of Bachelor of Medicine:—*Second Class.*—W. M. Bergin, A. S. Cobbledick, J. J. S. Lucas.

**Royal College of Surgeons.**—First Professional Examination for the Diploma of Fellow—W. R. Battye, F. H. Scrase.

**Examining Board in England by the Royal Colleges of Physicians and Surgeons** [Conjoint Board].—*Elementary Anatomy*—T. Aubrey, C. O. Bodman, A. Coleridge, S. L. Compton, J. B. S. D'Aguilar, J. R. Frost, C. A. H. Gee, C. B. Horsburgh, F. P. Hughes, C. W. W. James, A. R. McEnnery, C. H. Pring, P. G. Stock, P. W. White, S. R. Williams. *Elementary Biology*—C. O. Bodman, S. L. Compton, H. Hemsted, C. B. Horsburgh. *Chemistry*—W. N. Blatchford, C. A. H. Gee.

Second Examination.—E. V. Foss, R. F. Moorshead, W. R. Battye. *Anatomy only.*—C. M. Mitchell, F. C. Morgan.

Final Examination.—*Surgery*—*W. M. Willis, *G. B. Price, D. S. Gerrish. *Midwifery*—G. B. Price, E. W. Ormerod, D. S. Gerrish. *Medicine*—F. W. Cave.

**Society of Apothecaries.**—Preliminary Examination. Part II.:—*Materia Medica*—J. Freeman. *Surgery*—E. R. Bowen. *Midwifery*—J. H. R. Pigeon.

BRISTOL ROYAL INFIRMARY.

Thomas Carwardine, M.D., M.S. Lond., F.R.C.S. Eng., has been appointed House Surgeon, and Harold F. Mole, F.R.C.S. Eng., L.R.C.P. Lond., House Physician.

Deputy Surgeon-General J. F. Shekleton, M.D., has retired from the office of Secretary and House-Governor after nearly eight years service following a long and active membership of the Committee. Mr. Edward A. Leonard has been appointed his successor.

BRISTOL GENERAL HOSPITAL.

G. B. Price, M.R.C.S., has been appointed Casualty Medical Officer.

* Denotes completion of Examination.

